# Recovering *The Principles of Humane Experimental Technique*: The 3Rs and the Human Essence of Animal Research

**DOI:** 10.1177/0162243917726579

**Published:** 2017-08-28

**Authors:** Robert G. W. Kirk

**Affiliations:** 1 Centre for the History of Science, Technology and Medicine, Faculty of Biology, Medicine and Health, University of Manchester, Manchester, UK

**Keywords:** academic disciplines and traditions, arts and aesthetics, epistemology, ethics, expertise, politics, power, governance

## Abstract

The 3Rs, or the replacement, reduction, and refinement of animal research, are widely accepted as the best approach to maximizing high-quality science while ensuring the highest standard of ethical consideration is applied in regulating the use of animals in scientific procedures. This contrasts with the muted scientific interest in the 3Rs when they were first proposed in *The Principles of Humane Experimental Technique* (1959). Indeed, the relative success of the 3Rs has done little to encourage engagement with their original text, which remains little read and out of print. By adopting a historical perspective, this article argues that one explanation for this disjunction may be found in another, more celebrated, event of 1959: C. P. Snow’s Rede lecture on *The Two Cultures*. The moral outlook of *The Principles of Humane Experimental Technique* derived from an earlier ethos wherein humanistic and scientific values occupied a shared culture. While the synthetic style of *The Principles* has hindered its readership, this article concludes that there is value to recovering the notion that the humanities and social sciences can contribute to the improvement of animal research.

## Introduction

Today, the 3Rs, or the replacement, reduction, and refinement of animal research, have become established worldwide as *the* ethical approach to governing animal-dependent science. In spite of the rapid rise of the 3Rs to recognition and implementation from the 1990s, few are familiar with their history. Indeed, a curious characteristic of the 3Rs is that one needs apparently to know nothing of their history in order to follow their precepts. Far from being required reading, the original text in which the 3Rs were introduced, *The Principles of Humane Experimental Technique* (W. M. S. Russell and Burch 1959), is out of print as it has been, almost continuously, since publication of the first editions by Methuen & Co. in 1959 (UK) and Charles C. Thomas in 1960 (United States).^1^ This article adopts a historical perspective to explain this incongruity through reference to the ways in which relations between scientific and human values have changed over time within predominantly British or British-influenced culture. It concludes by considering implications for animal research today.

Contemporary commentators who have engaged with *The Principles* tend to report that text to be unclear and confusing, noting that the multiple definitions of the 3Rs today depart in significant ways from their original formulation (e.g., Tannenbaum and Taylor Bennett 2015). One explanation for this, explored by this article, is that *The Principles* is shaped by a historically specific moral outlook, which assumed that a shared set of values united work across the “sciences” and “humanities.” Readers will be well aware this view is less prevalent today, indeed *ST&HV*’s aims and scope state that over time “more and more, human values come into conflict with scientific advancement.” Even at the time of writing, harmonious relations between humanities and the sciences were waning as the latter increasingly challenged the former for prestige, power, and societal leadership. In sum, *The Principles* is a difficult text to read because it is grounded in a specific formulation of scientific humanism that lent itself to a complex and eclectic multidisciplinary style and approach that has little traction today.

The notion that human and scientific values apparently conflict, which motivates *ST&HV* and drives the ongoing controversy around animal research, assumes and perpetuates a historically bounded distinction: that modern intellectual society is divided into the “two cultures” of the sciences and humanities.^2^ Similarly, it can often seem to follow that humanism and the sciences operate with distinct knowledge forms, the former evaluative the latter factual. In contrast, *The Principles* assumes the very opposite: that the humanities and sciences reinforce each other and would continue to do so into the future through their shared values and broadly shared epistemology. This latter is rooted in a specifically Victorian scientific humanism described by Smith (2003) as a “common context” wherein “scientists believed that the practice of science itself, with its openness to truth, was a model for good citizenship” (p. 237). Central to Victorian and Edwardian moral values was the notion of individual character, which, within the life sciences as in wider culture, operated as the ethical guarantor of scientific behavior, providing moral justification for scientific research as “biology explains character, and that character, or the capacity for individuality, is both the key top social advance and an ethical ideal” (Smith 2003, 178). Although *The Principles* is coauthored by W. M. S. Russell and Rex Burch, it was Russell, an Oxford trained biologist and polymath, who is responsible for the style, form, and content of the text. Russell was the son of the marine biologist Sir Frederick Stratten Russell through whom he would have been exposed to and embedded within the scientific humanism framework that dominated the elite intellectual culture of late nineteenth- to mid-twentieth-century Britain.

That this distinctive moral outlook was already fading by the 1950s and has lost all traction today can explain why *The Principles* was and remains a challenging text to understand. For instance, a contemporary reader may well be perplexed as to why *The Principles* gives no consideration whatsoever to the views and concerns of the lay public (cf. Hobson-West, this volume). However, such an approach follows logically from the “common culture” of interwar Britain that carefully balanced a commitment to social modernization through science with a conservative resistance to mass society (cf. Smith 2003, 236-38). As we shall see, these values allowed scientists “to present themselves as both special, a legitimate part of the traditional elite, and democratic, through their contribution to the well-being of all” (Smith 2003, 236).^3^ Such an outlook precluded the value of democratic “inclusion” within the practice or governance of science, a view that shaped not only *The Principles* but also the broader work of the Universities Federation for Animal Welfare (UFAW), which funded the work.

This article is premised on the claim that the original formulation of the 3Rs can only be properly understood in the context of a scientific humanism that was inherited from the Victorian period and already in sharp decline at the time of their constitution. In part 1, the origins of the *The Principles* and the 3Rs are located within an effort to mobilize the common culture of humanities and the sciences to establish a science of animal welfare during the mid-twentieth century. Part 2 reviews a tension at the heart of the common culture that led to a schism in which humanities and sciences famously came to be understood as two cultures. Finally, part 3 examines how the common culture that sustained synthesized moral and scientific epistemology of *The Principles* stood in tension with the growing schism between the humanities and the sciences in the wake of the two cultures. In concluding, a case for the continued relevance to animal research of understanding the historical background of the 3Rs is presented.

## Making a Science of Animal Welfare—The Common Context in Action

In the interwar period, the UFAW, a self-styled “scientific” animal advocacy organization, worked to establish animal welfare as an applied science. UFAW was committed to scientific meliorism: the ethos that science was the best route to improving society that derived from the common context of scientific humanism. The British Science Guild, for instance, saw itself as a politically and ideologically neutral body existing to promote the application of science to the improvement of society (MacLeod 1994). Prior to UFAW, no systematic efforts had been made to mobilize scientific meliorism to improve the place of animals in society. Animal advocacy, as then, was focused less on “improving” the lot of animals than preventing cruelty toward them (e.g., Beers 2006). In practice as much as in rhetoric, animal advocacy was political, framed by a discourse that historians have shown to be shaped as much by human concerns for nation, gender, class, and race than for the animal in and of itself (Ritvo 1989; Kean 1998). Interwar animal advocacy was widely perceived to be opposed to science largely through its association with antivivisectionism, which by the close of the nineteenth century had effectively polarized scientific and social values among those committed to the cause. In what remains the most comprehensive analysis of the late nineteenth-century British vivisection controversy, French (1975) concluded that “[a]ntivivisectionists foresaw the cold, barren, alienation of a future dominated by the imperatives of technique and expertise. It was not experiments on animals they were protesting, it was the shape of the century to come” (p. 412). Antipathy to scientific values, technique, and expertise was characteristic of early twentieth-century antivivisection, which constrained the possibility for scientific meliorism to take hold within this wider animal advocacy movement. It was in response to this problem that Charles Hume, Honorary Secretary of the British Science Guild, established the University of London Animal Welfare Society (ULAWS) in 1926.

Like the Guild, which served as inspiration and model, ULAWS was founded on the premise that science, being free of emotion and sentiment, was better placed to improve society than democracy and public debate alone. Hume believed scientists, veterinarians, and the elite intelligentsia were all hampered from acting to improve the welfare of animals through fear of being tarnished by association with the rampant emotionalism of radical animal advocates and antivivisectionists. ULAWS, renamed the UFAW as it grew from a local group to national network of university based branches, adopted a distinctive position within the animal advocacy movement by refusing to invoke the politics of mass emotionalism. Rather than appealing to the public, UFAW aimed to educate the educators by appealing “to scientific men in our universities to be generous enough to devote some part of their thought and effort to means for diminishing the hardships to which animals are subjected in their contacts with human civilization” (Hume 1939, 39). In the spirit of democratic elitism characteristic of scientific humanism, UFAW sought to harness scientific expertise and directly apply it to the improvement of “animal welfare.” Hitherto, animal advocacy had tended to focus on the prevention of cruelty rather than the promotion of “welfare.” The latter tended to be used to capture an existential but still political condition, explicit, for instance, in the naming of the National Council for Animals’ Welfare. UFAW, in contrast, approached animal welfare as an “object” that could be scientifically identified, measured, manipulated, and improved.

Rather than intervening upon the traditional landscape of emotive public appeals and political campaigns, UFAW focused on applying science to achieve demonstrable improvements to animal welfare. The focus on practical action as opposed to political rhetoric was reflected in the organization’s mission “to diminish, by methods appropriate to its special character as a university organization, the sum total of pain and fear inflicted by man on animals” (ULAWS 1934, 2). UFAW’s approach reframed animal welfare as a scientific rather than political object with the intention of moving animal advocacy away from political campaigning toward the development of animal welfare as a science. As such, UFAW pioneered a middle ground where scientists and veterinarians could deploy their respective expertise to improve the welfare of animals with “a maximum of sympathy but a minimum of sentimentality” (Hume [1962] 1982, 8). One consequence of this logic was that UFAW had no objection to the killing of animals. Their objective was to improve welfare by reducing suffering; thus a painless death was “humane.” Consequently, UFAW’s interwar activities involved enrolling veterinary, medical, biological, zoological, engineering, and related scientific expertise to establish humane techniques of killing animals, with a focus on stray animals (Vinter 1950), vermin (Wright 1936), and livestock (Hume 1927). Hume, for instance, having endorsed the electrical stunning of pigs (Hume 1935), became deeply skeptical of the dramatic uptake of electrocution as a humane method of killing in the wake of the Slaughter of Animals Act, 1933. In order to ascertain the humanity of electrocution for other animals, particularly dogs and cats, he worked with electrical engineers uncovering a method of determining whether a procedure was humane or cruel that had “appeared in an engineering journal and being scarcely intelligible to anybody but an electrical engineer or a physicist [had] been almost entirely ignored by veterinary surgeons and by lay humanitarians” (Hume 1939, 153). The example of electrocution is emblematic of UFAW’s vision for a science of animal welfare; it was less to be a discipline than an eclectic cross-disciplinary field. To this end, UFAW worked to connect disciplines that otherwise did not interact, seeking to catalyze collaborative approaches to problems that had escaped rigorous attention and could only be resolved by collaborative efforts across multiple forms of expertise.

UFAW worked to establish a culture of communication spanning science, society, and animal advocacy as well as expert specialisms that hitherto had not knowingly shared an object of interest. Establishing a science of animal welfare required the fostering of cross-disciplinary spaces of communication as

Opponents of the gin-trap must understand agriculture as well as trapping; the problem of cruel poisons is one for the chemist; the use of electricity both for killing and for immobilising animals raises questions on which the electrical engineer no less than the veterinary surgeon has a point of view; the mechanical engineer has already provided the separator as a partial solution of the problem of oil pollution, and the casting-pen and the pig trap for the slaughter-house; the zoologist, physiologist, and veterinarian, the theologian and the psychologist, the historian and the economist, have all some special knowledge bearing upon man’s relationship with animals. (ULAWS 1936, 164)

In arguing that the “study of animal welfare” should be “a department of sociology which has been neglected in its scientific aspect,” UFAW acknowledged that communication across disciplines had been a problem in itself (ULAWS 1936, 164). Nevertheless, making the study of “man’s dealings with the animals” a “scientific sociology” was a “task worthy of our universities and demands contributions from ethics and theology, economics, psychology, zoology, veterinary science, and various other branches of learning” (ULAWS 1931, 1). Such an inclusive academic vision tied to social progress reflected and embodied the interwar common context of a “world of values shared by scientists and non-scientific people…expressed in scientific thought and practice as well as in other walks of life” (Smith 2003, 212). Here, the plight of animals became both a collective agenda and a shared object of interest through which UFAW established a science of animal welfare through which specialists with otherwise diverse technical and scientific knowledge could come together.

UFAW’s interwar activities were diverse, addressing aspects of animal welfare from the humane slaughter of agricultural animals to the education of children in animal care. However, one critical area was absent: UFAW was “precluded by its constitution from engaging on either side in controversies relating to scientific experiments on animals” (ULAWS 1938, 167). Animal experimentation, or “vivisection” as it was still commonly known, proved a divisive subject that had threatened the viability of the society when established in 1926. Rather than serving as a platform upon which consensus and practical action could be cultivated to improve the welfare of animals, UFAW’s claim to be a scientific society for the promotion of animal welfare initially made it a target for all sides. Antivivisectionists and the scientific community each associated the new organization with their perceived opponents and attacked it accordingly. Only by adopting a strong noncommittal position was the society saved and the goodwill of scientists secured (Worden 1951). As a result of the initial vehemence against UFAW caused by the vivisection controversy, it remained silent on the question of laboratory animal welfare for a considerable time after it had established substantive credibility and trust within the scientific community. It was not until 1942 that the society felt sufficiently established to address the question, and even then it did so indirectly and with strategic care. This shift in stance was a response to concerns emerging from within the scientific community about the suitability of animals then available for research. Hitherto, animals had been procured for experimental research ad hoc from commercial breeders who were more interested in fancy, fur and pet trade than the needs of science. In 1942, a coalition of British scientific stakeholders called for government action to establish a systematic means for the production and provision of “standard” laboratory animals. Reluctantly, the Medical Research Council took responsibility for what was increasingly perceived as a threat to scientific research: the absence of healthy, reliable laboratory animals of known backgrounds (Kirk 2008). For UFAW, this presented an opportunity. By aligning the scientific need for *healthy* animals with the promotion of animal welfare, UFAW recasts its agenda to promote animal welfare as an essential component of reliable of animal research.

Importantly, UFAW’s point of entry was animal care *not* experimental practice per se. Helpfully, UFAW’s pragmatic raison d′être to reduce the “sum total” of suffering inflicted on animals by society allowed the organization to circumvent the contentious political question of the moral legitimacy of using animals for scientific purposes. Nevertheless, intervening in the practice of animal research was both a risky and challenging move for UFAW as an animal advocate organization (albeit a self-styled scientific one). Accordingly, UFAW strategically focused on practices of animal care rather than animal experimentation. This history is important because it reveals how the promotion of laboratory animal welfare and the recognition of the epistemological importance of care for science originated from a hierarchical division between labor in the “animal house” and the experimental work of the “laboratory.”

In the 1940s, the care and management of animals for scientific research remained largely unskilled and undervalued labor with little import for the actual work of scientific research beyond the provision of required resources. It was, in short, a safe target that allowed UFAW to promote laboratory animal welfare without any suggestion of an attempt to instruct scientists on how they should approach animal research. On the contrary, UFAW presented its work as intended to improve the extrascientific labor of the animal house so as to better serve the needs of animal research. Animal care was also a strategic target as it posed the familiar challenge of having been hindered in its development because knowledge and technical expertise were scattered across literatures and disciplines that lacked a shared position or concern to act as a cohesive gathering point. Through meetings and publications such as the landmark *UFAW Handbook on the Care and Management of Laboratory Animals* (Worden 1947), UFAW constituted a new space where diverse parties could work to improve laboratory animal welfare without challenging the legitimacy, authority, or credibility of animal research. In particular, the *UFAW Handbook* established itself as a standard text, positively reviewed within scientific literature as an “authoritative work” remarkable for having “persuaded such a large number of people to meet on a common ground of humanity and utility” (Elton 1948, 87).^4^ This work contributed to the professionalization of animal care, the emergence of the role of the “animal technician,” and the increasing transformation of the animal house from an ad hoc space to a highly regulated scientific environment analogous to the laboratory in postwar Britain. Consequently, the care and welfare of laboratory animals came to be recognized as integral to scientific epistemology and experimental practice. As historians and science studies scholars such as Asdal (2012) have persuasively argued, to properly understand science “contexts” should be seen as “integral to the very action” of performing science (p. 388). Arguably, the wider social, cultural, and political contexts surrounding the scientific use of animals should, then, be seen as integral to the transformation of animal care and experimental practices over time. This historical process might best be understood as the animal research nexus.

Importantly, UFAW’s strategic exploitation of the social and spatial separation of animal care and management from scientific research proper further entrenched the assumption that animal care and welfare concerned the animal house and not the laboratory. Research scientists had no objection to improving the work of the former, in large part because it made little obvious demand on the working practices of the latter. Indeed, the work of animal care and management could safely be ignored entirely, providing animals were available as research activities required them. Recognizing this, and buoyed by the success of the *UFAW Handbook*, UFAW initiated a second phase of work, which led to the 3Rs. In the first stage, interventions such as the *UFAW Handbook* (Worden 1947) located concern for the care and welfare of animals in the animal house. By improving husbandry and management practices, and embedding the promotion of animal welfare into the newly imagined role of expert animal technician, UFAW established concern for animal well-being as integral to, but a preliminary step away from, the experimental research in the laboratory. In the 1950s, UFAW turned its attention to experimental practice itself and the work of scientists proper, initiating the work that produced *The Principles of Experimental Technique* (1959). This strategic, social, and spatial distinction of the work of animal house and laboratory goes some way to explain why the language of care is almost entirely absent from *The Principles*. Instead, what came to be known as “humane experimental technique” established concern for the animal by grounding scientific practice within humanist values. *The Principles* was not, however, intended to be a work of moral philosophy: as the *UFAW Handbook* focused on the material labor of animal care, so too did *The Principles* address the practical work of experimental science. However, it did so in a number of ways not all of which coalesced into a cohesive and comprehensible study. For instance, very little systematic knowledge existed regarding the present or future needs of the animal the research community. Consequently, a preliminary step toward addressing animal welfare in the laboratory was to conduct what today would be seen as a social science investigation of animal research. UFAW’s vision for the development of humane experimental technique was, from the start, rooted in a common context that brought together perspectives and approaches from what are now separated across the humanities, social sciences, and life sciences.

In 1955, William Moy Stratton Russell and Rex Burch were employed to conduct this work, the former leading the project as “UFAW Research Fellow” and the latter acting as “field assistant,” conducting quantitative and qualitative mapping of animal research in the UK. Russell, a recent Oxford University zoology graduate, appeared ideally suited to UFAW’s cross-disciplinary ethos. An experienced animal researcher, Russell also possessed diverse interests that ranged across biological, psychoanalytic, behavioral, historical, and the sociological sciences. His synthetic approach was evident from the start as he embarked on a “historical study of the factors underlying the development and utilisation of new techniques in experimental biology…with special reference to changes in technique making for greater humanity.” Importantly, Russell considered “the power and precision of experimental methods…to be relevant to humanity, since they will tend to reduce the numbers of experimental animals required for obtaining results of a given accuracy.”^5^ This illustrated how social and scientific values were approached as one; integrated as opposed to separate, reflecting the ethos of the common context that shaped interwar intellectual life in Britain. Russell was a characteristic British interwar scientist, seamlessly weaving biological and cultural history into his narratives, producing synthetic accounts of evolutionary, societal, and human progress (cf. Smith 2003). Not only did this approach lend itself to an unproblematic synthesis of Russell’s study of the past and Burch’s focus on the present, but it also assumed without question that practical “factors making in general for increased humanity in the laboratory” were in large part social in character. As such, the improvement of animal welfare in experimental science for Russell required approaches and research techniques that today would be associated with the humanities and social sciences. While this interdisciplinary conjunction was retrospectively productive, eventually producing the 3Rs that are now widely established as ethical principles governing animal research, it equally hindered the intelligibility and reception of the ethos of humane experimental technique.

## The Two Cultures Context

As UFAW continued to work within the common context to develop coherent and pragmatic approaches to the promotion of laboratory animal welfare, by the late 1950s, the shared value system that fostered the seamless weaving of humanities, social, and life sciences approaches was beginning to unravel. Writing in 1956, the lapsed chemist and active novelist C. P. Snow (1956) lamented that a “separation between the two cultures has been getting deeper under our eyes; there is now precious little communication between them, little but different kinds of incomprehension and dislike” (p. 413). Snow described how the loss of a unitary intellectual culture produced a schism in which the humanities and sciences had become increasingly hostile and alienated from each other. In the British intellectual atmosphere of the 1950s, cultural authority was perceived to rest with the “traditional” academic disciplines of the humanities. However, the dominance of the humanities was threatened by a confident and expansive scientific culture. This scientific challenge to the cultural authority of the humanities, which was equally seen to be a challenge to humanist values, was what Snow famously named a schism of the two cultures.

Snow’s two cultures were not presented as a literal or essential incommensurable gulf between the humanities and sciences. Rather, he coined the term as a useful metaphor for a series of perceived sociocultural differences. Snow ([1959] 2014) acknowledged as much by describing the phrase as “more than a dashing metaphor, but a good deal less than a cultural map” (p. 9). One significant ambiguity was the place of the “social” sciences in Snow’s thesis. At the time, the social sciences had yet to establish a clear identity for themselves within the British academic system and were still relatively novel in American universities (Ross 2003). To the extent that Snow ([1963] 2014) acknowledged the social sciences at all he seemed to have in mind “social historians” who, forming a potential “third culture,” had discredited through rigorous scholarship the literary notion that science and technology had had only a deleterious effect upon society and its values (pp. 78-84). Nevertheless, Snow’s perceived schism within intellectual culture, shaped by social, political, economic, and intellectual concerns, turned most forcefully on the question of humane values. When Snow later presented his thesis as a public lecture in 1959, he initiated a vociferous debate that continues to this day (Ortolano 2009).

The two cultures was presented in the 1950s as a “new crisis in the universities” because within academia the “split seems to be deepen as each side blames the other” (Lovell 1959, 68). From the scientific perspective, the challenge was to obtain equity and authority within a university system long dominated by the humanities. From the humanist perspective, however, the institutionalization of the sciences had placed “universities in mortal peril” by eroding humanist values in favor of “scientific proofs: model satellites, and a dog martyred in a flying kennel” (Mansell Jones 1959, 11). The two cultures debate was shaped by social concerns over class makeup of the educated elite, access to economic resources, and the relative worth of disciplinary knowledge. The rhetoric of the two cultures placed at stake the authority to speak on the “human condition” and in doing so transformed the epistemological approach and ontological presumptions around the same. Snow’s initial 1956 presentation of the two culture schism addressed what was lost in the schism, which, in sum, was a fuller understanding of the human condition. Science was “driving down into the problems of will and cause and motive” and so “those who do not understand the [scientific] method will not understand the depths of their own culture” (Snow 1956, 413). Science was revealing a radically different portrait of what it was to be human than that which the humanities had traditionally assumed. Snow (1956) explained that where humanists had widely identified the human condition as fundamentally tragic the “impulse behind the scientists drives them to take nothing as tragic that can conceivably lie within men’s will” (Snow 1956, 413). Science offered “nothing but contempt” for the “defeatist” humanist view of the human condition. Tragedy, Snow observed, sustained a sociocultural politics of conservatism that served humanists whose vested interest was to maintain their cultural authority “somewhere near the top” (Snow 1956). In this early rendition of the two cultures thesis, Snow focused upon the intellectual and moral characteristics of humanists and scientists. He concluded that it was the “moral health of the scientists which, in the last few years, the rest of us have needed most; and of which, because the two cultures scarcely touch, we have been most deprived” (Snow 1956). In other words, the most damaging consequence of the two cultures schism was that it deprived society of the progressive moral values inherent in the sciences.

Yet it was the very values of science that humanists most detested. Or, at least, this became the case following the 1962 retort of the British literary critic F. R. Leavis in 1962. Leavis was affronted not so much by the two cultures thesis, but by the fact Snow had had the audacity to speak on the subject. For Leavis, Snow could have no claim to be a scientist or a humanist. To speak for either was a symptom of a technocratic intellectual decrepitude that derived from science and threatened British culture. Leavis ([1962] 2003) dismissed Snow’s naive commitment to social progress, demanding to know how one could isolate a “social condition” from the “individual condition” as the worth of “individual lives cannot be aggregated or dealt with quantitatively” (pp. 65-66). Responding to Snow’s laudatory account of the industrial revolution as the motor for societal improvement, Leavis cited Ruskin for whom:

well-being or welfare could not conceivably be matters of merely material standards of living, with the advantages of technology and scientific hygiene. And there we have the gap—the gap that is the emptiness beneath Snow’s ignorance—between Snow and not only Ruskin, but the great creative writers of the century before Snow: they don’t exist for him; nor does civilisation. (Leavis [1962] 2003, 70)

Civilization, which for Leavis translated as human worth or what he termed the “third realm,” was not found in abstract scientific concepts but rather the “collaborative-creative process…in the living present, in the creative response of individuals, who collaboratively renew and perpetuate what they participate in—a cultural community of consciousness” (Leavis [1962] 2003, 75). This third realm, of which for Leavis literature was the example par excellence, was neither private nor public but something else: what we might perhaps think of as a site of future-oriented creative collective becoming. Where Snow located “social hope” in the technocratic promise of science to improve societal conditions over generations, Leavis abhorred any suggestion that moral value could be reduced to and measured by the material improvement of a population. It was this intellectual climate in which *The Principles of Humane Experimental Technique* was developed and presented. As such, the soil was unlikely to be receptive to the seed.

## The Principles of the Humane Experimental Technique in the Context of the Two Cultures

Where Snow and Leavis saw two cultures in conflict, W. M. S. Russell retained a notion of a common culture, albeit one made up of distinctive ways of thinking and working that continuously demanded the labor of synthesis. *The Principles* did not directly engage the challenge of the two cultures, though the problem clearly influenced, albeit incoherently, Russell’s thinking on the challenge to communication posed by disciplinary specialism. Certainly, Russell’s adoption of cybernetics was driven by the hope it could provide a shared interdisciplinary language to overcome the splintering of disciplines into ever more specialist and divided areas. Specialism, for Russell, was the problem, and the solution was the cultivation of a common context. This can be discerned from a short-story he penned in response to a challenge set by *The Observer* to imagine life in 2500 AD.^6^ In Russell’s story, three brothers competed to retrieve a mysterious object, which won the right to marry the daughter of the “system coordinator” (the ruler of this highly rational future society). The first, Cathodus, was a distinguished physicist (i.e., scientist) and the second, Census, a leading socio-psycho-technologist (for which we might read sociologist and humanist). The third, Biophile, was in the view of the first two a failure who owed his existence to an emotional weakness in their father who should have followed societal convention in having him euthanized as a child. Biophile was uneducated, irrational, and unable or unwilling to use technology. He spent his time isolated from rational society seeking to learn to live in natural harmony with wild animals (an activity he excelled at though it had no societal value). As the story unfolded, Cathodus’s faith in technology was shown to be misplaced and Census’s expertise led him to repeatedly misjudge the alien cultures he encountered. Only Biophile progressed, rescuing his brothers en route, and eventually winning the prize and thus the hand of the system coordinator’s daughter. Biophile’s success was achieved through his collaboration with a range of animal species. Wealthy, respected, and married to a gifted wife, Biophile established the first academic study of “animal behavior,” instantiating intellectual respect for nontechnological knowledge of the natural world and establishing something akin to a common culture. The salient message, underlined by the disdain that the educated Cathodus and Census showed their father and brother, was that perceived cultural divides, whether between disciplines or intellectual and moral values, needlessly limited individual and collective ambition. Moreover, the pursuit of science and technology without recognition of their inherent humane value damaged society by eroding respectful interest in all life, human and animal alike.

An analogous valorization of synthesis framed *The Principles*, which dismissed the common misconception of “an irreconcilable conflict between the claims of science and medicine and those of humanity in our treatment of the lower animals” (W. M. S. Russell and Burch 1959, 3). Adopting the then-voguish discourse of cybernetics, which provided a synthetic interdisciplinary language attentive to fostering stability from chaotic systems via reflective “feedback,” Russell argued that “the humanist possible treatment of experimental animals, far from being an obstacle, is actually a prerequisite for successful animal experiment” (W. M. S. Russell and Burch 1959, 4). The core message—that a respectful interest in animals and their welfare was a necessary condition for scientific epistemology—was identical to that espoused by Biophile. In a sweeping move that was intended to establish a clear agenda Russell identified a need:

to create a new discipline of applied science. Now that specializations are multiplying with unheard-of rapidity, the creation of yet a new one may cause many hearts to sink; but this new science has the virtue of being a synthetic one, which brings together under a common view-point a vast variety of facts and ideas from a multitude of existing fields. (W. M. S. Russell and Burch 1959, 6)

The new discipline was that of humane experimental technique and it was presented as three principles, the replacement, reduction, and refinement of animal research, or the “3Rs.”

The 3Rs function as the pragmatic heart of *The Principles*, a point at which the project of humane experimental technique most clearly aligned with the successful “how to” pragmatism of the *UFAW Handbook*. This is not to say there was any attempt to meet the style of an instructional handbook. On the contrary, the 3Rs were presented conceptually, as Russell himself admitted “we have made no attempt to begin the cataloguing of special techniques…we have sought only to establish the general principles of this new subject” (W. M. S. Russell and Burch 1959, 6). The 3Rs were the “methods” and later “modes” of “diminishing inhumanity in experimentation” (W. M. S. Russell and Burch 1959, 7), which Russell took to be the goal of humane experimental technique. Diminishing inhumanity was the “criterion of humanity” (W. M. S. Russell and Burch 1959, 157). Humanity entailed a humane disposition toward animals premised upon kindness and benevolence, which in practice required action to diminish suffering and distress. Rather than being a tightly defined normative value, humanity was a general descriptive term, which evoked a specific comportment toward other forms of life that was at once humanistic and scientific. Russell attempted to establish humane objectively as the causing or diminishing of suffering in the other. This presumed that the experiential state of the animals could be known and measured. At the same time, he retained—even valorized—the commonsense association between humane and moral values. Rather than a disadvantage, the long-standing moral sense of humanity was important because it “reflects the fact that man surpasses all other species in his capacity for social-cooperation” (W. M. S. Russell and Burch 1959, 14). In this somewhat convoluted and certainly incoherent way, Russell sought to establish the characteristics of good scientific practice through a complex and (particularly to modern readers) mixture of scientific, social scientific, and humanist understandings of behavior that framed humane experimental technique as both a human performance and a collaboration across species.

Russell’s attempts to provide an objective understanding of humanity, without reducing complexity or evoking normative values, produced a wide-ranging and sometimes incoherent narrative style that preferred the addition of further layers of complexity over a progression toward clarity. For instance, a basic indicator of inhumanity was pain and fear in the animal, which collectively could be thought of as distress. Drawing on recent research on stress, Russell proposed that “anatomical responses to hormones are drastically affected by what might appear to be trivial factors disturbing the ‘peace of mind’ of the animal.”^7^ Russell’s point was that the social relation between human and animal directly affected animal physiology and as such had consequences for experimental practice. However, his mode of making this point progressed from demonstrable physiological responses to presumptions of animal consciousness. Attribution of consciousness to animals was highly controversial at this time because consciousness was thought to be outside the bounds of experimental scientific inquiry. Yet Russell was unworried by his presumption. Drawing on psychoanalysis, another fashionable form of knowledge (though not so fashionable that the average pharmacologist would be familiar with its finer aspects), Russell claimed that to deny “consciousness in non-human animals” betrayed a “pathological” psyche resulting from traumatic experiences in childhood (W. M. S. Russell and Burch 1959, 15). Psychoanalytic statements such as this, which uncritically integrated moral values, scientific knowledge, and psychosocial health, offered the reader little more than a Hobson’s choice: accept animal consciousness or reveal oneself to be psychologically unsound. In the absence of a scientific argument, Russell drew on psychoanalysis to pathologize a moral choice, a narrative style which held together only within the common context of scientific humanism.

Russell’s forays though social science, psychoanalysis, and moral philosophy were a disappointment to Hume, who struggled to follow the argument and had, in any case, envisaged a text more in the style of the *UFAW Handbook*: pragmatic, applied, and actionable. Hume was equally worried by Russell’s tendency to criticize as opposed to develop a positive case for reform. An early draft, possibly the first, read “it is somewhat remarkable that the realization of these facts by experimental workers (other than those specifically studying them) should be so slow and gradual; but so it is…and the laboratory is apparently the last stronghold of resistance to the unreserved acceptance.”^8^ Hume reminded Russell that as an animal welfare society, albeit one closely aligned with scientific culture, UFAW remained an “outsider” and so could not risk outright criticism pushing the potential reader to ask “who are you to tell me how to do my job?”^9^ By publication, this modest criticism had been replaced by a capitalized assertion that reference to humanity must “NOT BE TAKEN TO IMPLY ETHICAL CRITICISM OR EVEN PSYCHOLOGICAL DESCRIPTION OF PERSONS PRACTICING ANY GIVEN PROCEDURE” (W. M. S. Russell and Burch 1959, 14). If the humane treatment of animals was the guarantor of reliable scientific research, as *The Principles* argued, then to suggest that animals had not or were not being treated as such was to cast doubt on the value of scientific work past and present. Such an adversarial position was anathema to cause; it had to be assumed that science was sound and that scientists sought to treat animals humanely. While Russell attempted to subtly assert the need for further work on the technical aspects of learning to differentiate humane from inhumane practices, the absence of clear criticism allows *The Principles* to be read in the main as a vindication of existing animal research as opposed to a manifesto for changed practices.

Disregarding Hume’s concerns, Russell retained his complex interdisciplinary style and in the final chapter of *The Principles* marshalled cutting-edge approaches from the social sciences to further integrate humane, scientific, and psychiatric (psychoanalytic) values. Appropriating the recent work of Theodor W. Adorno, Else Frenkel-Brunswik, Daniel Levinson, and Nevitt Sanford of the University of California, Berkeley, which had identified the so-called authoritarian personality type as being most susceptible to fascism, Russell correlated the authoritarian personality with inhumane dispositions toward animals (C. Russell and Russell 1958). Further, he argued that the pathological “authoritarian” personality was incompatible with thinking “in terms of many variables” and thus was incompatible with science itself (C. Russell and Russell 1958, 154). Regardless of what we might make of such claims today, it is significant that humane experimental technique was premised on the assumption that scientific practice, moral values, and the psychological makeup of human and animal were presented as coconstituted and fundamentally integrated.^10^ It followed that humane concern for the animal was integral to both science and scientific identity. Humanist values were aligned with and assimilated within scientific epistemology. Good science demanded good conscience.

*The Principles* wove together the humanities, the social sciences, and the life sciences, shifting from history to psychoanalysis, through biochemistry, pharmacology, ethology, physiology, cybernetics, psychosomatics, and psychosocial health, often without explicit acknowledgment of the change in register. Russell’s shifting disciplinary perspectives were less an experiment in interdisciplinary writing than an exercise in transdisciplinarity. His writing freely took a concept from one discipline (say the biological phenomenon of heterosis) and used it to diagnose and pathologize a social phenomenon (such as the two culture schism) before reaching a quasi-moralistic conclusion (the two cultures schism is bad because it threatens academic vitality). Yet viewed through the lens of the common context such imaginative leaps were unexceptional. For scientific humanists, physiological knowledge of how biological life regulated, stabilized, and organized the individual appeared necessarily to have implications for how social life achieved the same for the group (Smith 2003, 219-20). Russell’s propensity to explain one discipline’s problem with another’s knowledge or method, for example, using a biological process to explain a historical trend, was made doubly obtuse by a tendency to draw on cutting-edge work that was not necessarily widely known within, never mind without, its respective discipline. Russell’s apparent assumption that the reader was not only a polymath, but one who was up to date with the very latest ideas across a huge range of fields troubled and frustrated Hume, who complained the narrative style was:

high-falutin’, complicated, obscure, and too long winded. The references to psychoanalysis are of great interest to psychoanalysts, but hardly interesting to readers who have no knowledge of psychoanalysis, who will be in the majority.^11^

Hume had a point. Such was the challenge of communicating across disciplines; one had to be familiar with, if not an expert in, each specialism to recognize the importance of each point and how the combination constructed a whole. Few animal-dependent scientists would have been familiar with psychoanalysis, the scientific credentials of which were much disputed. Yet psychoanalytic concepts provided the core justification for Russell’s claim that humanist values, scientific epistemology, and a healthy psyche were integral guarantors of good animal research.

Russell’s determination to transcend disciplinary boundaries and interlace distinct disciplinary knowledges, which included the physiological science of Ivan Pavlov, the ethological studies of Desmond Morris, and the sociohistorical lessons of classical history (Cleisthenes being a favored source when writing against the two cultures schism), demanded too much of the target audience: animal-dependent scientists. The latter were further distanced by Russell’s preference for theoretical as opposed to practical approach. In any case, the attempt to locate humanist values at the heart of scientific practice was antithetical to the wider climate. Against the context of the two cultures schism, scientists had little interest in humanism and humanists distrusted science as having little to no meaningful value. A prominent review in *Nature* concluded that *The Principles* was

not sufficiently informative to be used as a guide to details of experimental design or to husbandry of experimental animals. Perhaps its chief purpose is to stimulate thought on both of these topics, and it is to be hoped that it will succeed in doing so. (Weatherall 1959, 1676)

More problematically, the strategic decision to shy away from explicit critique allowed *The Principles* to be read as endorsing the status quo. If humanity were an epistemological condition for the possibility of reliable science, then necessarily it followed that science had to meet this criteria, as evidentially the vast majority of published science was reliable science. Where, then, was the need for reform? This was the message taken by most scientific readers of *The Principles*. One reviewer, for instance, concluded that the book made difficult reading and would likely be “left on the shelf” only to be taken down on occasion to respond to antivivisectionist criticism (Anon 1959).

## Conclusions: The Two Cultures as a Challenge to a Culture of Care

Historians of science and medicine have emphasized the importance of understanding context less as an explanatory device and more as an integral part of science (Asdal 2012). At the same, the language of common context (Smith 2003) or common culture (Anderson 2014) has been invoked as a means to understand how social, political, economic, and cultural values are embedded in knowledge, whether such knowledge is derived from the natural sciences, social sciences, humanities, or a composite (such as *The Principles*). Reading *The Principles* today, we might conclude that it lends support to the argument that the humanities and social sciences have value when aligned with the natural sciences or that they can make meaningful contributions to laboratory animal research. The contrast between the rise to prominence of the 3Rs as an ethical framework for animal research in recent years and the relative lack of interest in *The Principles* both at publication and today illustrates both the gains and risks of transdisciplinarity (working across several disciplines to produce coherent knowledge and approaches shared by the collective). As disciplinary constraints weaken new possibilities for asking and answering questions, the pathway to, and relevance for, a given audience can diminish in kind. Sensitivity to this tension, as much as to the importance of disciplinary language, would be critical to any endeavor to revive Russell’s vision that the humanities and social sciences have something positive to contribute—not only to understanding but also to the practice of animal research (cf. Davies et al. 2016).

As the sciences usurped the cultural authority of the humanities in twentieth-century Britain, the distinctive common context that shaped Russell’s world view broke up as the academic topology fractured into two cultures. As a result, *The Principles* failed to find an audience within the scientific community in large part because it was embedded within an ethos inherited from Victorian culture that had lost traction and meaning. In the absence of a common context, Russell’s eclectic transdisciplinary style, combined with the intent to establish humanist values at the heart of scientific epistemology, held little practical or political value to animal research. Why, then, have the 3Rs survived? One reason is that the principle of “replacement” offered a fresh approach to the antivivisectionist movement. A century of antivivisectionism campaigning had failed to make any tangible impact on the growth of animal research, which in Britain had risen from the hundreds to the millions by the 1960s. For antivivisectionists, the third “R” of replacement provided a practical as opposed to political approach to reversing the exponential growth of animal research. Moreover, working to replace animals in scientific research overcame the long-standing disagreement that had fractured the British antivivisectionist movement on the issue of gradual or immediate abolition. In the 1961, inspired by *The Principles*, the National Antivivisection Society, the British Union for the Abolition of Vivisection, and the Scottish Society for the Prevention of Vivisection established the Lawson Tait Memorial Trust to fund research into the development and promotion of “alternatives” to animal research.^12^ The goal of replacement, renamed alternatives, was taken up by antivivisectionism as a collective strategy to curtail animal research by funding the development of superior, nonanimal-based approaches to medical research. The antivivisectionist roots of the alternatives movement hindered the ability of early organizations to build trust and credibility with the scientific community. However, following the establishment of further organizations with less obvious ties to antivivisectionism—such as the Fund for the Replacement of Animal Research in 1969, which strategically appropriated scientific as opposed to antivivisectionist discourse—the 3Rs were eventually recovered and found an audience within the scientific community. For some, the 3Rs were the practical tools to bring about an abolitionist agenda, while for others, they stated in a formal sense what was implicit to animal research: a desire to reduce, refine, and, where possible, replace the use of animals. As such, the 3Rs became a useful ground to build a typical British compromise where a variety of otherwise opposed positions could coalesce and broadly agree to a way forward. However, in the process, the 3Rs lost their originary humanist roots to become rational procedures capable of aligning moral and scientific values within a pragmatic ethical framework.

Recovering the broader humanistic ethos that shaped *The Principles* invites reflection on the capacity of the humanities and social sciences to shape animal research. In one explicit retort to the separation of the humanities and sciences, Russell asserted:

The other great progressive human activity is art, which is so closely related to science as to be virtually the same activity. Thus it comes that the greatest scientific experiments have always been the most humane and the most aesthetically attractive, conveying that sense of beauty and elegance which is the essence of science at its most successful. (W. M. S. Russell and Burch 1959, 157)

Russell believed that the development of humane experimental technique would allow the “aesthetic aspect of experimentation” to “take its place among what are curiously and selectively called the humanities” (W. M. S. Russell and Burch 1959, 163). Here, perhaps, Russell has in mind an exploration of the affinities between the aesthetics and ethics of science. This was to act as a corrective to the incremental alienation of the humanities from sciences through specialization. In his deeply idiosyncratic style, Russell explained the danger of specialism by mobilizing biological phenomenon to reach sociological conclusions. While “harmless in itself,” specialism became harmful over time by inhibiting communication across fields, thereby causing “whole streams of science to come to halt for lack of what we might properly call hybrid vigour” (W. M. S. Russell and Burch 1959, 159). Here again, Russell invoked the notion that biological populations that bred only with themselves declined in vigor, productivity, and general health over time and applied it to specialism and, by extension, the two cultures schism. In Russell’s wider research on human behavior, the two cultures schism became an intellectual pathology attributed to a “neurotic taboo” that forbade “the exploration of human feelings and social interactions” (C. Russell and Russell 1957, 196). Integrating ethological, psychoanalytic, neuropsychological, and social sciences, alongside literary and historical argument, was the only appropriate response to challenge of the “scientific society” that must ensure “the development of social institutions which can oppose any permanent rigid specialization, while permitting maximal variance of behaviour” (C. Russell and Russell 1957, 197).

In writing *The Principles*, Russell explained that he “sought only to limn the barest of outlines” leaving it to others to “fill in the interior” and develop humane experimental technique (W. M. S. Russell and Burch 1959, 167). Today, the work of filling in the interior is well under way. Yet the relevance of *The Principles* to such work is not obvious unless the text is understood within its historical context. It was through reference to interiority—both human and animal—that humane experimental technique established its ethical guarantor. Moral values were not to be imposed upon the scientist from without via social pressure or law but rather were embodied in the very epistemology and practice of science. Russell mobilized scientific humanism to embed ethical behavior within the individual personality of the scientist. By investing moral values in scientific identity, Russell placed the scientist as knowing subject with as much at stake in the practice of experimental research as the animal subject. Within human experimental technique, cross-species epistemological cooperation was elided with that of ethical codependency: to safeguard their identity as human and scientist, the researcher had to care for the animal. While Russell chose not to develop or articulate the moral and practical importance of his notion of cross-species cooperation in any pragmatic or systematic sense, he did present it as an area of critical importance.

For instance, one suggested criterion for assessing the humanity of a procedure was “that of the animal’s behaviour *toward the experimenter*” (W. M. S. Russell and Burch 1959, 32, emphasis original). This acknowledges that any successful culture of care is subjective, situated, and contingent. As such, humane experimental technique and the 3Rs were never intended to be institutionalized. Rather, they were to be internalized: embodied in the human scientist and enacted in scientific research.

Drawing on the ethologist Konrad Lorenz, Russell insisted that experimentalists must ensure their animal “subjects” possessed *mens sana in corpore sano*, a healthy mind in a health body, noting that we “will not get the one without the other” (W. M. S. Russell and Burch 1959, 13). This established a clear obligation toward domesticated animals that:

have lost many of their original responses, and suffered disruption of a formerly well-organized and dove-tailed behaviour system, in connection with their long history in a new kind of environment, one in which many of their needs may be supplied by man…We have often to supplement their behaviour, for we are now an essential part of their world.” (W. M. S. Russell and Burch 1959, 32)

As the animals’ environment was a “man-made ecology,” the question of animal welfare was less of “an intrinsic problem, as those of wild animals are to some extent, but a problem of human sociology; for they are determined by human needs and decisions” (W. M. S. Russell and Burch 1959, 32-33). In the laboratory encounter, human and animal were codependent: shaped and shaping each other. The salient difference being that environmental factors within the laboratory were more open to human control. “[S]cience,” Russell wrote, was “indissolubly linked to the social activity of co-operation, which will find its expression in relation to other animals no less than to our fellow humans” (W. M. S. Russell and Burch 1959, 157). Once it is accepted that the experimental animals’ world is in large part a human construction, then it follows that the humanities and social sciences have a role to play in improving animal research and welfare.

## Notes

1. In 1990, marking the renewal of interest in the 3Rs (replacement, reduction, and refinement), Universities Federation for Animal Welfare (UFAW) reissued a small-run facsimile edition, and in 2009, a much abridged version retitled *The Three Rs and the Humanity Criterion* was made available by Fund for the Replacement of Animals in Medical Research.

2. Smith (2003) persuasively makes this point by illustrating how the natural and biological sciences are excluded from accounts of British intellectual culture.

3. The style, form, and content of *The Principles* can largely be attributed to the biologist and later polymath W. M. S. Russell who, through his father the marine biologist Sir Frederick Stratten Russell, would have been exposed to the world of values that shaped elite intellectual culture from the late nineteenth to the mid-twentieth century as outlined by Smith (2003).

4. In contrast, the antivivisectionist community responded negatively to UFAW’s move to improve the welfare of laboratory animals. For instance, a lead editorial in *The Abolitionist* argued “an antivivisectionist may not be a supporter of societies that exist solely for the welfare of animals” yet it is not easy “to understand why many of those who support these societies are not antivivisectionists.” This was a direct reproach to UFAW, whose work had been tolerated when “its representatives remained neutral,” but now UFAW acted “in a way detrimental to our cause” it was taken to have sided with science (Anon 1944).

5. W. M. S. Russell, “Draft Letter of Introduction for Burch” c. July 1956, W. M. S. Russell Papers, folder WCR 1/1/1 (hereafter WMSR). I thank Cleo Paskal for access to these private papers maintained at the University of Nottingham, UK.

6. Russell’s story was shortlisted from 2,240 entries, anonymized, and presented with two others for readers to vote for the best. Russell came second, with 3763 votes, the winning story had been written by the novelist E. L. Malpass polling 3,802 votes. The third story, with 2,337 votes, had been written by Brian Aldiss who later became one of Britain’s foremost science fiction writers.

7. WMSR, “Scientific Investigations. Humane Experimental Technique in the Laboratory Brief Progress Report,” December 10, 1954, WCR 1/2/6.

8. WMSR, WCR 1/2/5, 5–6.

9. WMSR, “C W Hume Attachment Commentary on Russell’s Letter of Introduction for Burch. 16th November 1954,” WCR 1/1/1.

10. WMSR, “Scientific Investigations,” 1954.

11. WMSR Letter Hume to W. M. S. Russell, August 20, 1957, WCR 1/1/4.

12. The original trust deeds survive at British Union for the Abolition of Vivisection Archive DBV[2]/28/26.
